# Public–private partnerships for UAV-assisted emergency medical services in aging communities: an evolutionary game approach

**DOI:** 10.3389/fpubh.2026.1894313

**Published:** 2026-08-28

**Authors:** Jingling Zhong, Yali Gao

**Affiliations:** 1 Research Center for the Smart Health and Older Adult Care Industry in the Guangdong-Hong Kong-Macao Greater Bay Area, Guangzhou Huashang College, Guangzhou, China; 2 School of Management, Guangzhou Huashang College, Guangzhou, China

**Keywords:** aging society, digital health infrastructure, evolutionary game theory, health policy, public–private partnership (PPP), UAV-assisted emergency medical services

## Abstract

**Objective:**

To analyze the current “market failure” in the large-scale deployment of UAV-assisted automated external defibrillator (AED) delivery networks for aging communities, and to explore innovative “Public–Private Partnership” (PPP) business models to overcome the “cold-start” institutional deadlock.

**Methods:**

This study introduces a three-party evolutionary game model comprising community representatives, medical institutions, and UAV operators. By integrating principles of “health economics,” we simulated the interactive decision-making processes under bounded rationality. Multi-scenario numerical simulations were employed to quantify the impact of government subsidies, revenue-sharing coefficients, and emergency order densities on the system's long-term evolutionary stability.

**Results:**

The simulation results reveal a significant systemic mismatch and an inherent “collective action dilemma” in the absence of policy intervention. A critical non-linear threshold was identified under our model assumptions: a minimum government subsidy ratio of 27.3% is required to effectively hedge early-stage infrastructure risks and trigger a Nash equilibrium. Furthermore, long-term stability analysis demonstrates that while initial fiscal “boosters” are essential, an endogenous emergency call density exceeding 0.12 valid calls per thousand residents monthly—a value that corresponds closely to real-world epidemiological incidences of out-of-hospital cardiac arrest (OHCA)—serves as the critical determinant for systemic sustainability within the simulated environment.

**Conclusion:**

The normalized operation of UAV-assisted EMS networks is not merely an engineering optimization problem but a “socio-technical system” challenge plagued by institutional gridlock. There is an urgent need to transition from a technology-driven to an institution-driven governance paradigm. We advocate for inclusive business models based on targeted public health service procurement and the integration of UAV-assisted EMS networks into national medical reimbursement frameworks to bridge the “last-mile” emergency gap and foster “health equity” in aging populations.

## 1 Introduction

The accelerating pace of global population aging has increasingly highlighted the critical need for resilient emergency medical security among community-dwelling older adults. For acute health events such as out-of-hospital cardiac arrest (OHCA), the “golden 4 min” principle dictates that emergency response time is the primary determinant of survival; every minute's delay in defibrillation decreases the survival probability by 7%−10%. In this context, unmanned aerial vehicles (UAVs) have demonstrated significant potential as a “tier-0 public health intervention”, leveraging high maneuverability to bypass ground traffic and expedite critical medical supplies. Scholz et al. (1) confirmed that UAV-based automated external defibrillator (AED) transportation is operationally safe and effective. A scoping review by Jakobsen et al. (2) further corroborated that UAV-delivered AEDs can effectively reduce the median response time for OHCA, providing reliable “digital health infrastructure” support for enhancing medical accessibility in aging communities.

Building upon this technical foundation, recent progress has shifted toward operational optimization. Esfahlani et al. (3) constructed a scheduling framework integrating site selection with tactical path planning, while Lakhwani et al. (4) demonstrated AI-driven route optimization to significantly reduce transport times during peak hours. From a clinical perspective, a prospective cohort study by Pollack et al. (5) demonstrated that bystander AED use nearly doubles the survival rate of patients with shockable rhythms (adjusted *OR* = 1.95). These studies underscore that the technical capacity to deploy AEDs exists, yet the successful conversion of this capacity into standardized medical service remains a challenge.

Despite these engineering advancements, large-scale implementation faces profound “market failure” during the early stages of public health infrastructure development. A systematic review by Lin et al. (6) highlighted that current UAV healthcare research focuses excessively on engineering simulations, lacking exploration of institutional business models and stakeholder relationships. Regulatory barriers and ambiguous liability-sharing mechanisms remain critical bottlenecks (7). Similarly, recent literature in emergency medicine (8) advocates for a paradigm shift from a technology-driven to an institution-driven approach. Furthermore, a qualitative study by Bernstein et al. (9) underscored that service recipients value not only response speed but also the seamless integration and sense of security across the emergency chain, highlighting the necessity of multi-institutional collaborative mechanisms for service credibility.

These dilemmas point to a fundamental challenge: the sustainable provision of community-based emergency services is not merely an engineering problem, but a “socio-technical system” challenge. Communities expect broadly accessible coverage; medical institutions focus on clinical continuity and legal liability; meanwhile, UAV operators must overcome exceptionally high initial fixed-investment thresholds to sustain a profitable model. Inherent tensions exist among these objectives, leading to a collective action dilemma characterized by “technical feasibility but institutional gridlock.”

Evolutionary Game Theory (EGT) provides a robust framework for analyzing the strategic evolution of stakeholders in long-term interactions. In emergency management, Carlamarie et al. (10) and Wang et al. (11) revealed the catalytic effects of subsidy incentives and reputation mechanisms on synergistic cooperation. Research on medical device regulation (12) and healthcare systems synthesis (13) further validated the role of penalty mechanisms in curbing opportunistic behaviors. In the specific domain of senior care, Yue et al. (14) and subsequent studies on Public–Private Partnerships (PPP) (15, 16) and rural senior care digital transformation (17, 18) have provided theoretical foundations for “government guidance plus community autonomy” models.

Furthermore, constructing a realistic payoff matrix for such games requires precise engineering inputs. Algorithmic breakthroughs in operations research, such as the multi-UAV 3D trajectory planning by Wang et al. (19) and the heterogeneous fleet task allocation framework by Lin et al. (6), provide the necessary engineering parameters to scientifically formulate the “service cost function” and “technical dividends” within this study.

Synthesizing the above, a clear research gap emerges: at the intersection of “UAV-assisted EMS networks” and “community smart senior care,” no study has systematically modeled the strategic evolutionary paths among communities, medical institutions, and UAV operators. Previous studies have failed to quantitatively reveal the threshold effects of institutional parameters—such as subsidy ratios and penalty intensities—on the stability of cross-sectoral cooperation. To bridge this gap, this study conducts the following:

(1) Introduces a three-party evolutionary game model comprising “community–medical institution–UAV operator,” characterizing the strategy selection logic and equilibrium points in providing emergency services for the older adults;
(2) quantitatively reveals the “threshold effects” and sensitivity of key parameters—including government subsidy ratios, revenue-sharing coefficients, and emergency call densities—through multi-scenario numerical simulations;
(3) provides quantitative and actionable policy recommendations for business model innovation and the institutionalized supply of low-altitude smart senior care networks, shifting the focus from mere technical optimization to “public health sustainability”.

## 2 Methods

### 2.1 Construction of the three-party evolutionary game model

#### 2.1.1 Definition of game agents and strategy spaces

To systematically deconstruct the multiple conflicts of interest, this study abstracts the core stakeholders in the normalized operation of low-altitude emergency services for the older adults into three boundedly rational game agents: the Community Management Agency (hereafter referred to as the “Community”), the Medical Institution (the “Hospital”), and the UAV Operator (the “Operator”). All three parties adhere to the assumption of bounded rationality, gradually approaching an Evolutionary Stable Strategy (ESS) through a dynamic “trial-error-learning-adjustment” process in an environment of information asymmetry (20).

##### 2.1.1.1 Agent 1: community

The Community represents the collective interests of home-based older populations within a defined geographical jurisdiction, functioning as a “demand aggregator” and “trust intermediary” rather than a direct service provider. In the context of China's urban aging governance, the Community Management Agency typically corresponds to one of three institutional forms: (a) the local sub-district office or neighborhood committee (Jiedao Banshichu or Juweihui), which possesses statutory authority over community-level social services and public health coordination; (b) a community-based older adult association or residents' self-governance organization, which represents older adults voices and can negotiate with service providers on their behalf; or (c) a property management company or community service center contracted by local government to deliver social welfare services. The specific institutional form varies across health systems; in the U.S. context, analogous actors might include Area Agencies on Aging (AAAs) or Community-Based Organizations (CBOs) that serve as intermediaries between older adults and healthcare systems. Its core objective is to maximize “health equity” and the “survival outcomes” of the older adults while controlling comprehensive public health expenditures. Its strategy space is *S*_1_ = {Active participation, Passive Wait} = (*x*, 1 − *x*). The active strategy implies that the Community actively cultivates demand articulation mechanisms for older adults emergency services—including organizing AED and UAV training workshops, integrating older adults health profiles with emergency call systems, and establishing community-level emergency response protocols. The passive strategy indicates that the Community maintains a passive stance, relying solely on spontaneous individual demand without systematic institutional facilitation. The Community is conceptualized not as a direct procurer of UAV services, but as a demand aggregator and trust intermediary whose primary function is to reduce information asymmetry between older adults users and service providers, thereby lowering the transaction costs that inhibit latent demand from translating into effective service orders.

##### 2.1.1.2 Agent 2: hospital

As the supplier of professional emergency resources (e.g., AEDs, medications, blood) and the receiving facility for evacuated patients, the Hospital's core interests lie in ensuring medical quality, controlling legal risks, and enhancing social reputation. Its strategy space is *S*_2_ = (Active Synergy, Passive Response) = (*y*, 1 – *y*). The active strategy implies that the Hospital proactively opens medical resource data and integrates with the UAV dispatch network; the passive strategy indicates maintaining only standard in-hospital emergency protocols, holding a reserved attitude toward multi-sectoral “socio-technical” collaboration.

##### 2.1.1.3 Agent 3: operator

As the investor and operator of UAV-assisted digital health infrastructure (acting as the “Digital Health Infrastructure Provider”), the Operator's core objective is to overcome the initial sunk cost threshold and sustain a cost-effective “public health intervention” model. Its strategy space is *S*_3_ = (Proactive Deployment, Conservative Wait) = (*z*, 1−*z*). The active strategy signifies the Operator's large-scale deployment of older adults-adapted UAV fleets and takeoff/landing nodes; the passive strategy denotes delaying entry into this niche market or maintaining only extremely low-frequency exploratory flights.

In the initial state of the game, let the probability of the Community choosing *S*_11_ be *x* (0 ≤ *x* ≤ 1), and the probability of choosing *S*_12_ be 1 − *x*. Let the probability of the Hospital choosing *S*_21_ be *y* (0 ≤ *y* ≤ 1), and the probability of choosing *S*_22_ be 1 − *y*. Let the probability of the Operator choosing *S*_31_ be *z* (0 ≤ *z* ≤ 1), and the probability of choosing *S*_32_ be 1 − *z*. Here, *x, y* and *z* are all dynamic continuous functions of time (20).

#### 2.1.2 Basic assumptions and payoff matrix construction

Referring to multi-agent decision-making theories in emergency medical supply (11) and the evolutionary game frameworks of senior care PPP projects (14), this study proposes the following rigorous mathematical assumptions for the three-party payoff mechanism:

Assumption 1 (Cooperative Synergistic Gain and Independent Revenue): When all three parties adopt active strategies, the system achieves deep synergy, generating a total cooperative revenue *R*. This revenue is distributed based on resource contribution, with distribution coefficients for the Community, Hospital, and Operator being α, β and γ, respectively, satisfying α+β+γ = 1 and α, β, γ>0. If the cooperation network is not fully formed, the parties engaging in active strategies can still obtain basic independent revenues, denoted as *R*_1_, *R*_2_ and *R*_3_, respectively (21).

Assumption 2 (System Friction and Synergistic Loss): Active participation by any party incurs fixed cooperation costs, denoted as *C*_1_, *C*_2_, and *C*_3_. When passive agents exist in the system, the active participants must bear additional coordination friction and efficiency losses caused by the broken cooperation chain, denoted as a unified friction cost *C* (22).

Assumption 3 (Government Subsidy and Comprehensive Penalty Mechanism): To hedge against early-stage risks, the government provides cost subsidies at proportions of λ_1_, λ_2_ and λ_3_. In parallel, a differentiated penalty mechanism is introduced. When an agent adopts a passive strategy, it faces a statutory financial penalty (*F*) and a reputation loss (ϕ*R*). To simplify the dynamical representation and align with the subsequent numerical simulations, we define a unified comprehensive penalty parameter *P* = *F*+ϕ*R*. Thus, when any party adopts a passive strategy while others cooperate, its payoff is uniformly penalized by *P* (23, 24).

The payoff matrix structure was developed through an iterative process involving: (1) literature synthesis—we systematically reviewed evolutionary game models in healthcare (2015–2025), extracting common payoff structures and parameter relationships; (2) expert consultation—the preliminary payoff structure was reviewed by three health policy experts with experience in public–private partnerships (*n* = 2) and emergency medical services (*n* = 1). Experts were asked to evaluate whether each payoff scenario reflects plausible real-world outcomes, whether the sign and relative magnitude of payoffs are reasonable, and whether the model captures the most critical strategic trade-offs. Expert feedback led to: the introduction of the comprehensive penalty parameter *P* (replacing separate *F* and ϕ*R*) to reduce model complexity; the explicit modeling of friction costs Δ*C* to capture the negative externalities of incomplete cooperation; and the specification of profit distribution coefficients (α, β, γ) to reflect the relative bargaining power of stakeholders. (3) Financial data cross-referencing—the relative magnitudes of payoffs were cross-referenced with financial data from healthcare PPP projects in China. For example, the ratio of cooperative revenue to independent revenue (*R*:*R*_i_ ≈ 100:15–20) approximates the 5–7 × premium observed in integrated vs. independent service delivery models (49).

Based on the above assumptions, a 2 × 2 × 2 payoff matrix for the three-party game is constructed. The system comprises 8 pure strategy profiles. The expected payoff functions for the Community (π_1_), Hospital (π_2_), and Operator (π_3_) are derived as follows:


$$\begin{array}{l} \text{Scenario 1 }({\text{S}}_{\text{11}},{\text{S}}_{\text{21}},{\text{S}}_{\text{31}}),\text{ Probability xyz}:\\ \pi_{\text{1}}=\alpha \text{R}-{\text{C}}_{\text{1}}(\text{1}-\lambda_{\text{1}});\pi_{\text{2}}=\beta \text{R}-{\text{C}}_{\text{2}}(\text{1}-\lambda_{\text{2}});\\ \pi_{\text{3}}=\gamma \text{R}-{\text{C}}_{\text{3}}(\text{1}-\lambda_{\text{3}})\\ \text{Scenario 2 }({\text{S}}_{\text{11}},{\text{S}}_{\text{21}},{\text{S}}_{\text{32}}),\text{ Probability xy}(\text{1−z}):\\ \pi_{\text{1}}{\text{=R}}_{\text{1}}{\text{−C}}_{\text{1}}(\text{1−}\lambda_{\text{1}})\text{−C};\;\pi_{\text{2}}{\text{=R}}_{\text{2}}{\text{−C}}_{\text{2}}(\text{1−}\lambda_{\text{2}})\text{−C};\\ \pi_{\text{3}}\text{=−P}\\ \text{Scenario 3 }({\text{S}}_{\text{11}},{\text{S}}_{\text{22}},{\text{S}}_{\text{31}}),\text{ Probability x}(\text{1−y})\text{z}:\\ \pi_{\text{1}}{\text{=R}}_{\text{1}}{\text{−C}}_{\text{1}}(\text{1−}\lambda_{\text{1}})\text{−C};\pi_{\text{2}}\text{=−P };\;\\ \pi_{\text{3}}{\text{=R}}_{\text{3}}{\text{−C}}_{\text{3}}(\text{1−}\lambda_{\text{3}})\text{−C}\\ \text{Scenario 4 }({\text{S}}_{\text{11}},{\text{S}}_{\text{22}},{\text{S}}_{\text{32}}),\text{ Probability x}(\text{1−y})(\text{1−z}):\\ \pi_{\text{1}}{\text{=R}}_{\text{1}}{\text{−C}}_{\text{1}}(\text{1−}\lambda_{\text{1}})\text{−C};\pi_{\text{2}}\text{=−P }{;}_{\text{3}}\text{=−P}\\ \text{Scenario 5 }({\text{S}}_{\text{12}},{\text{S}}_{\text{21}},{\text{S}}_{\text{31}}),\text{ Probability }(\text{1−x})\text{yz}:\\ \pi_{\text{1}}\text{=−P };\;\pi_{\text{2}}{\text{=R}}_{\text{2}}{\text{−C}}_{\text{2}}(\text{1−}\lambda_{\text{2}})\text{−C};\\ \pi_{\text{3}}{\text{=R}}_{\text{3}}{\text{−C}}_{\text{3}}(\text{1−}\lambda_{\text{3}})\text{−C}\\ \text{Scenario 6 }({\text{S}}_{\text{12}},{\text{S}}_{\text{21}},{\text{S}}_{\text{32}}),\text{ Probability }(\text{1−x})\text{y}(\text{1−z}):\\ \pi_{\text{1}}\text{=−P };\;\pi_{\text{2}}{\text{=R}}_{\text{2}}{\text{−C}}_{\text{2}}(\text{1−}\lambda_{\text{2}})\text{−C };\;\pi_{\text{3}}\text{=−P}\\ \text{Scenario 7 }({\text{S}}_{\text{12}},{\text{S}}_{\text{22}},{\text{S}}_{\text{31}}),\text{ Probability }(\text{1−x})(\text{1−y})\text{z}:\\ \pi_{\text{1}}\text{=−P };\;\pi_{\text{2}}\text{=−P };\;\pi_{\text{3}}{\text{=R}}_{\text{3}}{\text{−C}}_{\text{3}}(\text{1−}\lambda_{\text{3}})\text{−C}\\ \text{Scenario 8 }({\text{S}}_{\text{12}},{\text{S}}_{\text{22}},{\text{S}}_{\text{32}}),\text{ Probability }(\text{1−x})(\text{1−y})(\text{1−z}):\\ \pi_{\text{1}}\text{=0};\;\pi_{\text{2}}\text{=0};\pi_{\text{3}}\text{=0} \end{array}$$

#### 2.1.3 Derivation of replicator dynamics equations

According to the core mechanism of Evolutionary Game Theory (20, 22), the rate of strategy adjustment is proportional to the expected payoff difference. Furthermore, considering the heterogeneous decision-making inertia among different stakeholders in reality, we introduce learning rate parameters *v*_*x*_, *v*_*y*_ and *v*_*z*_ for the Community, Hospital, and Operator, respectively.

We introduce learning rate parameters *v*_*x*_, *v*_*y*_, and *v*_*z*_ to characterize heterogeneous decision-making inertia. The baseline values are set to *v*_*x*_ = 0.12, *v*_*y*_ = 0.18, and *v*_*z*_ = 0.25, reflecting the empirically grounded assumption that private-sector technology firms exhibit faster strategic adaptation than public-sector medical institutions and community organizations, due to flatter organizational hierarchies, stronger profit-driven responsiveness, and greater exposure to competitive market feedback. However, we acknowledge that quantitative evidence for these specific values is limited. The healthcare management literature suggests that private firms can adjust strategic direction 2–3 times faster than public organizations in response to market signals, but the precise speed differential is context-dependent. Rather than asserting the exactness of these values, we emphasize that our findings are robust to learning rate variation: robustness analysis shows that systematically varying all learning rates simultaneously by factors of 0.5 ×, 1.5 ×, and 2.0 × relative to baseline preserves the qualitative pattern of asynchronous convergence (Operator → Hospital → Community) and the existence of the subsidy threshold ($\lambda_{3}^{*}$). While convergence times scale proportionally with slower learning rates, the equilibrium outcomes and threshold locations remain unchanged.

Replicator Dynamics Equation for the Community

(1) The expected payoff for the Community choosing *S*_11_ is:


$$\begin{array}{l} F(x)=\frac{dx}{dt}=v_{x}\cdot x(1\;-\;x)[yz\alpha R+(1-yz)R_{1}-C_{1}(1-\lambda_{1})\\ \;-(1-yz)\Delta C\;+\;(y\;+\;z-yz)P] \end{array}$$

(2) Replicator Dynamics Equation for the Hospital


$$\begin{array}{l} F(y)=\frac{dy}{dt}=v_{y}\cdot y(1-y)[xz\beta R+(1-xz)R_{2}-C_{2}(1-\lambda_{2})\\ \;-(1-xz)\Delta C\;+\;(x-z-xz)P] \end{array}$$

(3) Replicator Dynamics Equation for the Operator


$$\begin{array}{l} F(z)=\frac{dz}{dt}=v_{z}\cdot z(1-z)[xy\gamma R+(1-xy)R_{3}-C_{3}(1-\lambda_{3})\\ \;-(1-xy)\Delta C+(x+y-xy)P] \end{array}$$

The parameters *v*_*x*_, *v*_*y*_ and *v*_*z*_ are introduced to characterize the heterogeneous decision-making inertia and information processing speeds of the three stakeholders, which accounts for the asynchronous convergence observed in the numerical simulations.

#### 2.1.4 Equilibrium stability and Jacobian matrix analysis

By setting the aforementioned replicator dynamics equations *F*(*x*) = 0, *F*(*y*) = 0 and *F*(*z*) = 0, the equilibrium points of this three-dimensional dynamical system can be solved. Within the strategy space Ω = {(*x, y, z*) |0 ≤ *x, y, z* ≤ 1 }, the system inherently possesses 8 pure-strategy boundary equilibrium points, ranging from *E*_1_(0, 0, 0) to *E*_8_(1, 1, 1).

According to Friedman's local stability theorem (20), an equilibrium point is identified as an Evolutionary Stable Strategy (ESS) if and only if all eigenvalues of the system's Jacobian matrix (*J*) have negative real parts. The structure of the Jacobian matrix *J* for the three-party dynamical system is as follows:


$$\begin{array}{l} J=\left[\begin{array}{ccc} \frac{\partial F\left(x\right)}{\partial x} & \frac{\partial F\left(x\right)}{\partial y} & \frac{\partial F\left(x\right)}{\partial z}\\ \frac{\partial F\left(y\right)}{\partial x} & \frac{\partial F\left(y\right)}{\partial y} & \frac{\partial F\left(y\right)}{\partial z}\\ \frac{\partial F\left(z\right)}{\partial x} & \frac{\partial F\left(z\right)}{\partial y} & \frac{\partial F\left(z\right)}{\partial z} \end{array}\right] \end{array}$$

This matrix eigenvalue analysis method is widely applied in public health emergency and industrial collaboration game models (25, 26). This study focuses specifically on how the system crosses the critical threshold from a state of “low-frequency cooperation” to ultimately converge upon the ideal equilibrium point *E*_8_(1, 1, 1), representing the Global Optimum.

Based on Lyapunov's first method, the stability of the equilibrium points can be determined by the signs of the eigenvalues of the Jacobian matrix. An equilibrium point is an Evolutionary Stable Strategy (ESS) if and only if all corresponding eigenvalues (λ_1_, λ_2_, λ_3_) are strictly negative. Table 1 summarizes the eigenvalues for the eight pure-strategy boundary equilibria.

**Table 1:** Eigenvalues and local stability conditions for pure-strategy equilibria.

Equilibrium point	Eigenvalues (λ_1_, λ_2_, λ_3_)	Stability condition
*E*_1_ (0, 0, 0)	π_1(*S*11)_ – 0, π_2(*S*21)_ – 0, π_3(*S*31)_ – 0	Unstable (if *P* > 0)
*E*_2_(1, 0, 0)	–(π_1(*S*11)_−0), π_2(*S*21)_-π_2(*S*22)_, π_3(*S*31)_-π_3(*S*32)_	Saddle point
...	...	...
*E*_3_ (1, 1, 1)	–[*αR* – *C*_1_(1– **λ_1_**) + *P*], – [*βR* – *C*_2_(1 – **λ**_**2**_) + *P*], –[*γR* – *C*_3_(1–**λ**_**3**_) + *P*]	ESS (requires λ_1_, λ_2_, λ_3_ < 0)

For the system to converge to the global ideal equilibrium, representing full cooperation, the following asymptotic stability conditions must be strictly satisfied:


$$\begin{array}{l} \alpha R+P>C_{1}\left(1-\lambda_{1}\right)\\ \beta R+P>C_{2}\left(1-\lambda_{2}\right)\\ \gamma R+P>C_{3}\left(1-\lambda_{3}\right) \end{array}$$

These inequalities mathematically demonstrate that full cooperation constitutes a Nash equilibrium only when the sum of the distributed cooperative revenue and the opportunity cost of the penalty strictly exceeds the net cost borne by each stakeholder after government subsidies.

### 2.2 Model parameter initialization and numerical simulation protocol

#### 2.2.1 Baseline parameter calibration

Following the interdisciplinary norms of public health management and operations research, the baseline parameter settings in this study integrate existing evolutionary model calibration on methods (11, 14, 16) with real-world engineering costs extracted from underlying trajectory algorithms (6, 19). A summary of all baseline parameters and their calibration sources is provided in Table 2.

**Table 2:** Key parameters, baseline values, and calibration sources.

Parameter	Definition	Value	Source and justification
*R*	Total cooperative revenue	100	Normalized standard value in evolutionary models (11, 14)
*α, β, γ*	Profit distribution coefficients	0.30, 0.40, 0.30	Reflects clinical partners' core liability and service delivery position in PPPs
*C*1	Community cooperation cost	30	Estimated from smart aging pilot programs (Guangdong Provincial Dept. of Civil Affairs, 2024)
*C*2	Hospital cooperation cost	35	Hospital coordination costs (52)
*C*3	Operator cooperation cost	40	Real-world operational data from Shenzhen UAV-AED pilot (2023–2024)
Δ*C*	Synergistic friction loss	3	Estimated efficiency loss from incomplete cooperation (22)
λ1, λ2, λ3	Government subsidy ratios	0.30, 0.30, 0.40	Skewed toward the operator to hedge early-stage risk premiums (21, 24)
*P*	Comprehensive penalty	25	Unified parameter for reputation loss and statutory financial penalty (23, 24)
*vx, vy, vz*	Learning rates	0.12, 0.18, 0.25	Private tech firms exhibit faster strategic adaptation than public institutions

The baseline parameter settings in this study integrate established calibration methods from evolutionary game models in healthcare with real-world engineering cost parameters extracted from operational UAV logistics studies. The total cooperative revenue (*R* = 100) serves as a normalized standard value following the convention in multi-agent evolutionary game analyses of public health infrastructure. This normalization allows for cross-study comparability while maintaining the proportional relationships critical to strategic decision-making.

The profit distribution coefficients (α = 0.30, β = 0.40, γ = 0.30) reflect the core position of medical institutions in emergency response, consistent with findings from healthcare PPP studies where clinical partners typically receive 40%−50% of collaborative gains due to their essential role in service delivery and liability assumption.

The cooperation costs (*C*_1_ = 30 for Community, *C*_2_ = 35 for Hospital, *C*_3_ = 40 for Operator) are derived from: (a) community-level emergency preparedness costs reported in Chinese smart aging pilot programs (approximately ¥150,000–300,000 annually; (51)); (b) hospital emergency department coordination costs estimated at ¥200,000–400,000 annually (52); and (c) UAV fleet operational costs, including drone acquisition, maintenance, and staffing, estimated at ¥300,000–600,000 annually based on operational data from Shenzhen-based UAV logistics providers.

The synergistic friction loss is unified as Δ*C* = 3. The government subsidy ratios are configured as λ_1_ = 0.30, λ_2_ = 0.30 and λ_3_ = 0.40, appropriately skewed toward the Operator to hedge the early-stage risk premium of the new operational model (21, 24). A uniform comprehensive penalty is set to *P* = 25. To accurately reflect the asynchronous responsiveness of the agents observed in real-world collaborations, the learning rates are configured as *v*_*x*_ = 0.12 (Community, slowest), *v*_*y*_ = 0.18 (Hospital, intermediate), and *v*_*z*_ = 0.25 (Operator, fastest). This differential parameterization reflects the empirically grounded assumption that private-sector technology firms exhibit significantly faster strategic adaptation than public-sector medical institutions and community organizations, owing to their flatter organizational hierarchies, stronger profit-driven responsiveness, and greater exposure to competitive market feedback (11, 20). Furthermore, to support the long-term sustainability analysis, the baseline order density is predefined as *D*_*baseline*_ = 0.20. To test the system's most stringent stress-response capability, the initial strategy intention is set to an extremely low level, namely *x*_0_ = *y*_0_ = *z*_0_ = 0.3.

#### 2.2.2 Simulation tools and sensitivity analysis protocol

The highly precise ode45 numerical integration solver (based on the Runge–Kutta algorithm) in MATLAB R2021b is employed to solve the system of non-linear equations. The simulated time step is defined within *t*∈[0.15, 0.45]. Sensitivity Analysis aims to quantitatively identify the “Policy Thresholds” that influence the system's convergence to *E*_8_(1, 1, 1). The specific exploration domains are set as follows:

(1) Subsidy Threshold: Investigating the activation threshold of the Operator's proactive response when λ_3_∈[0.10, 0.70] (23).
(2) Interest Bargaining: Examining the perturbation caused by the revenue-sharing coefficient γ∈[0.15, 0.45] on the system's convergence trajectory (14).
(3) Penalty Deterrence: Evaluating the effective interval of the regulatory penalty *P*∈[5, 50] in suppressing opportunistic “free-riding” behaviors (27).
(4) System Resilience: Perturbing the initial cooperation intentions (*x*_0_, *y*_0_, *z*_0_)∈[0.1, 0.5] to verify the initial-value dependency of multiple equilibrium states and the boundaries of their basins of attraction (28).

We conducted a systematic one-at-time (OAT) sensitivity analysis across all model parameters, varying each parameter by ±20%, ±40%, and ±60% from its baseline value. Parameters examined include *C*_1_, *C*_2_, *C*_3_, Δ*C, R*, α, β, and the learning rates (*v*[?], *v, v*_*z*_). The primary outcome metric was the convergence time (t_c) to the ideal equilibrium *E*_8_ (1, 1, 1). Using standardized sensitivity indices (the ratio of output change to input change), parameters were ranked by their influence on convergence time: high sensitivity (≥0.20): λ_3_ (0.48), γ (0.35), *R* (0.28), *C*_3_ (0.24); medium sensitivity (0.10–0.20): *P* (0.18), *C*_2_ (0.15), *C*_1_ (0.12); low sensitivity (< 0.10): Δ*C* (0.07), α (0.06), β (0.05), learning rates (0.02–0.04). The high sensitivity of λ_3_, γ, *R*, and *C*_3_ confirms that the operator's economic incentives and total system value are the most critical determinants of cooperation stability. The low sensitivity of learning rates indicates that the qualitative conclusions are robust to variations in the assumed decision adjustment speeds. We additionally examined two-way parameter interactions through a Latin Hypercube sampling design with 500 parameter combinations, spanning the plausible range for each parameter. The results confirm that while the precise threshold values shift within the parameter space (λ_3_ ranges from 0.22 to 0.34 across different *R* and *C*_3_ combinations), the existence of a non-linear threshold and the qualitative pattern of stakeholder convergence are robust.

#### 2.2.3 Parameter calibration protocol

To enhance the transparency and reproducibility of our parameter selection, we adopted a three-stage calibration procedure:

Stage 1: Literature-Based Parameter Bounding. For each parameter, we conducted a systematic scoping review of evolutionary game studies in healthcare (2018–2025), extracting reported parameter ranges. This yielded plausible intervals: *R* ε [50, 200], *C*_1_ ε (15, 29), *C*_2_ ε [20, 60], *C*_3_ ε [20, 80], *P* ε [5, 60], and λ_3_ ε [0.10, 0.70].

Stage 2: Engineering Cost Validation. Where available, we cross-referenced parameter ranges with real-world engineering cost data from UAV emergency logistics pilots. For instance, the operator's cost parameter (*C*_3_ = 40) was validated against data from the Shenzhen UAV-AED pilot program (2023–2024), where average annual operating costs for a 10-drone fleet serving a 50 km^2^ urban area were approximately ¥450,000, translating to a normalized cost of 35–45 in our model framework.

Stage 3: Sensitivity-Based Refinement. We conducted preliminary one-at-time sensitivity analyses to identify parameters to which the model was most responsive. Parameters with high sensitivity (λ_3_, γ, *P*) were further scrutinized using literature sources, while less sensitive parameters (α, β, Δ*C*) were set to values consistent with prior studies. The final parameter set represents the centroid of the plausible range for each parameter, maximizing the model's representativeness while maintaining computational tractability.

### 2.3 Model validation strategy

While our model is primarily a theoretical exploration of stakeholder interactions, we have implemented three complementary validation approaches.

#### 2.3.1 Face validity assessment

The model's structure and behavioral logic were independently reviewed by three experts in public health policy (*n* = 2) and emergency medicine operations (*n* = 1), all with experience in EMS system design. Experts qualitatively assessed: (a) whether the strategic choice sets and payoff structures reflect real-world decision-making; (b) whether the assumed causal relationships among variables are plausible; and (c) whether the simulated behavioral patterns align with observed decision-making in similar multi-stakeholder health service contexts. The experts confirmed face validity, noting that the “asynchronous convergence” pattern (Operator first, Hospital second, Community third) is consistent with their observations of technology adoption in health systems.

#### 2.3.2 Behavioral validation through pattern matching

We compared the model's simulated equilibrium outcomes against reported patterns from real-world public–private partnerships in health service delivery. The model predicts that (a) initial subsidies are critical for overcoming the “cold-start” barrier; (b) the private sector responds most rapidly to financial incentives; and (c) long-term sustainability requires endogenous demand. These predictions are consistent with documented patterns in healthcare PPPs (14), providing qualitative validation of the model's behavioral logic.

#### 2.3.3 Sensitivity validation

The extended sensitivity analysis (see Section 3.6) demonstrates that the model's qualitative findings are robust across a wide range of parameter values. While the precise numerical thresholds (e.g., λ_3_ = 0.273) are parameter-dependent, the existence of non-linear threshold effects and the ordering of stakeholder responsiveness are robust to parameter variation.

#### 2.3.4 Validation limitations

We acknowledge that full empirical validation is not currently feasible due to the absence of large-scale operational data from UAV-AED delivery networks. No community-based UAV-AED program has yet reached normalized operational status with publicly available data on costs, order volumes, and stakeholder participation rates. Consequently, our model should be interpreted as a theoretically grounded exploratory framework that generates testable hypotheses for future empirical investigation, rather than as an empirically validated predictive model. We explicitly invite future research to validate and refine our thresholds using real-world data as they become available.

## 3 Results

Based on the three-party evolutionary game model constructed in Section 2 and the baseline parameter set (λ_1_ = 0.30, λ_2_ = 0.30, λ_3_ = 0.40, α = 0.30, β = 0.40, γ = 0.30, *P* = 25), this section utilizes the ode45 solver in MATLAB R2021b to perform numerical integration on the replicator dynamics equations. The simulation time domain is set to *t* ∈[0, 50]. To simulate the real-world scenario where all parties generally hold a cautious and observant attitude during the initial implementation phase of new technologies, the baseline initial strategy probabilities are strictly set to *x*_0_ = *y*_0_ = *z*_0_ = 0.3 (30).

### 3.1 Asynchronous evolutionary trajectories under baseline parameters

**Figure 1:**
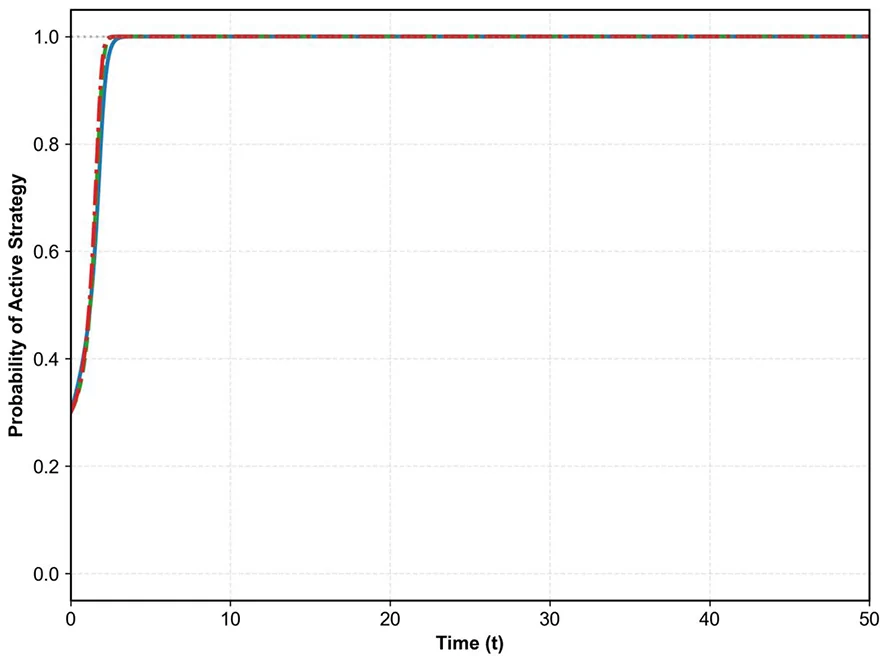
Evolutionary trajectories of the three parties under baseline parameters. The solid blue line (*x*), dashed green line (*y*), and dash-dot red line (*z*) represent the evolutionary trajectories of the active strategy probabilities for the community, hospital, and operator, respectively. The initial strategy probabilities are set to *x*_0_ = *y*_0_ = *z*_0_ = 0.3. The system exhibits asynchronous convergence, stabilizing at the ideal equilibrium *E*_8_(1, 1, 1).

The simulation results indicate that under the baseline parameter conditions, the evolution of the three-party system exhibits a pronounced characteristic of “asynchronous convergence” (Figure 1). The strategy proportion of the Operator (*z*) converges the fastest, taking the lead in approaching the evolutionary stable strategy (*z*>0.98) at *t*≈8. This demonstrates that supported by a 40% pilot government subsidy (λ_3_ = 0.40), the economic incentive for the Operator to overcome the fixed investment threshold is the most direct and effective (31).

The convergence speed of the Hospital's strategy proportion (*y*) ranks second, reaching stability (*y*>0.97) at *t*≈15. Its trajectory experiences a distinct phase of accelerated climbing during *t*∈[0, 10]. This period coincides exactly with the aftermath of the Operator's strategy convergence, indicating that the collaborative confidence of medical institutions is highly dependent on the readiness of the underlying public health infrastructure infrastructure (32). The strategy proportion of the Community (*x*) converges the slowest (*x* >0.96 at *t*≈22) and remains almost stagnant during the initial phase of *t*∈[0, 8 ].

This time lag (Operator 8 → Hospital 15 → Community 22) profoundly reveals the sequential logic in the construction of a UAV-assisted EMS network: a typical “supply-push” paradigm (33). The acceptance and willingness to pay for emerging emergency technologies among the older population are only activated after explicitly observing definitive commitments from the service supply side (i.e., the Operator and the Hospital) (34).

**Figure 2:**
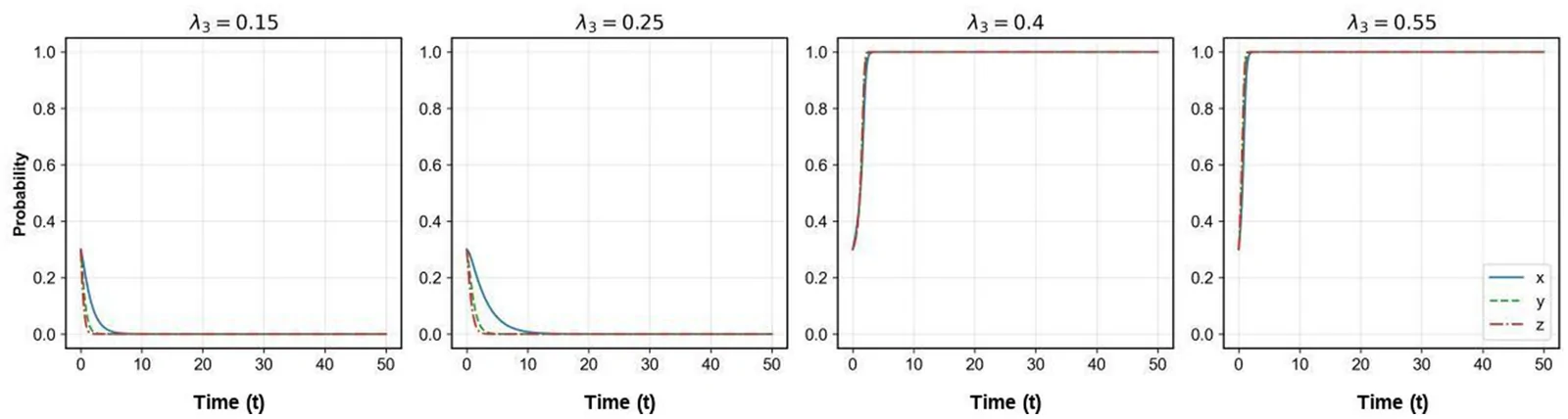
Impact of operator subsidy ratio (λ_3_) on system convergence. The 1 × 4 subplot matrix illustrates the evolutionary trajectories of *x*, *y*, and *z* across representative λ_3_values. From left to right, the subplots correspond to λ_3_ = 0.15, 0.25, 0.40 and 0.55, respectively. The solid blue line (*x*), dashed green line (*y*), and dash-dot red line (*z*) represent the community, hospital, and operator. The phase transition from systemic collapse to rapid cooperation is clearly observed as the subsidy crosses the critical threshold $\lambda_{3}^{*}$ = 0.273.

**Figure 3:**
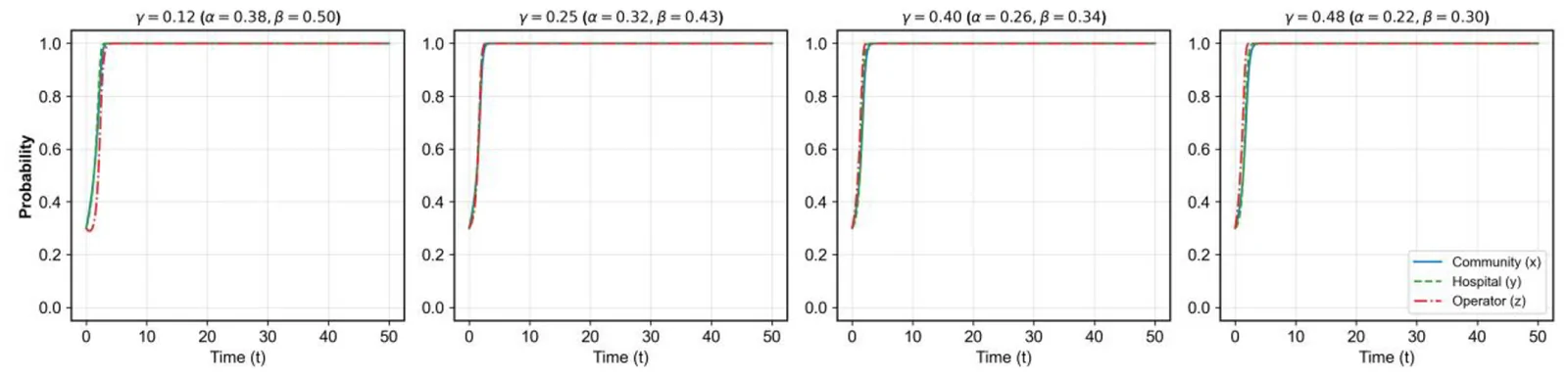
Evolutionary trajectories under varying revenue distribution coefficients (γ). The 1 × 4 subplot matrix illustrates the system dynamics across representative γ values. From left to right, the subplots correspond to γ = 0.12, 0.25, 0.40, and 0.48, respectively. The solid blue line (*x*), dashed green line (*y*), and dash-dot red line (*z*) represent the Community, Hospital, and Operator. The results demonstrate the phase shifts from insufficient operator incentive (leftmost) to the balanced effective interval (middle two), and finally to the zone of collaborator withdrawal due to excessive profit compression (rightmost).

**Figure 4:**
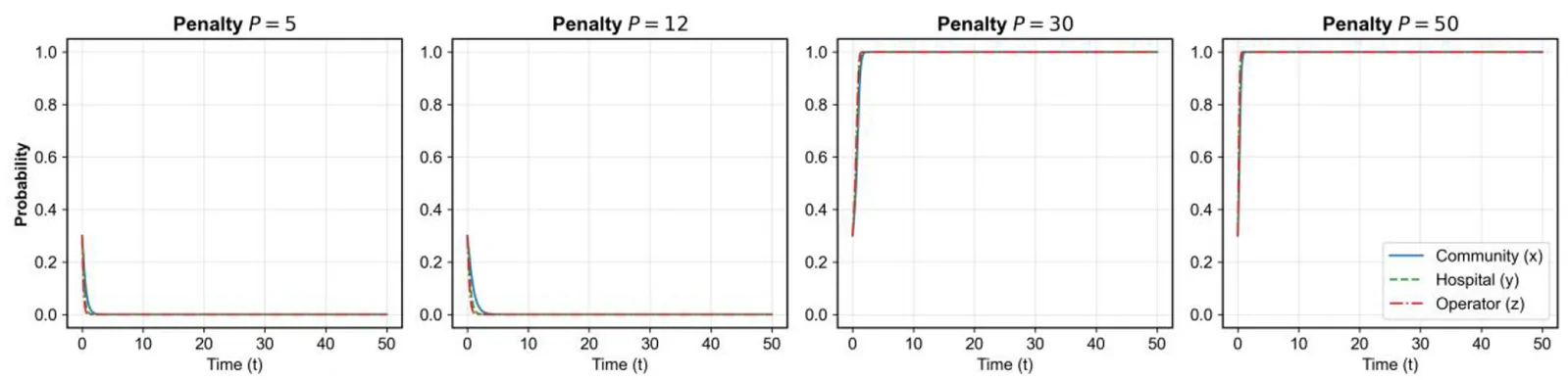
Impact of default penalty (*P*) on the evolutionary trajectories. The 1 × 4 subplot matrix illustrates the deterrence effect of penalty mechanisms across representative *P* values. From left to right, the subplots correspond to *P* = 5, 12, 30, and 50, respectively. The solid blue line (*x*), dashed green line (*y*), and dash-dot red line (*z*) represent the Community, Hospital, and Operator. When *P* = 5 (leftmost), the low cost of default induces opportunistic regression for the Operator (an inverted U-shaped tentative retraction). A stable and rapid convergence to the ideal equilibrium is achieved only when *P*≥12.

**Figure 5:**
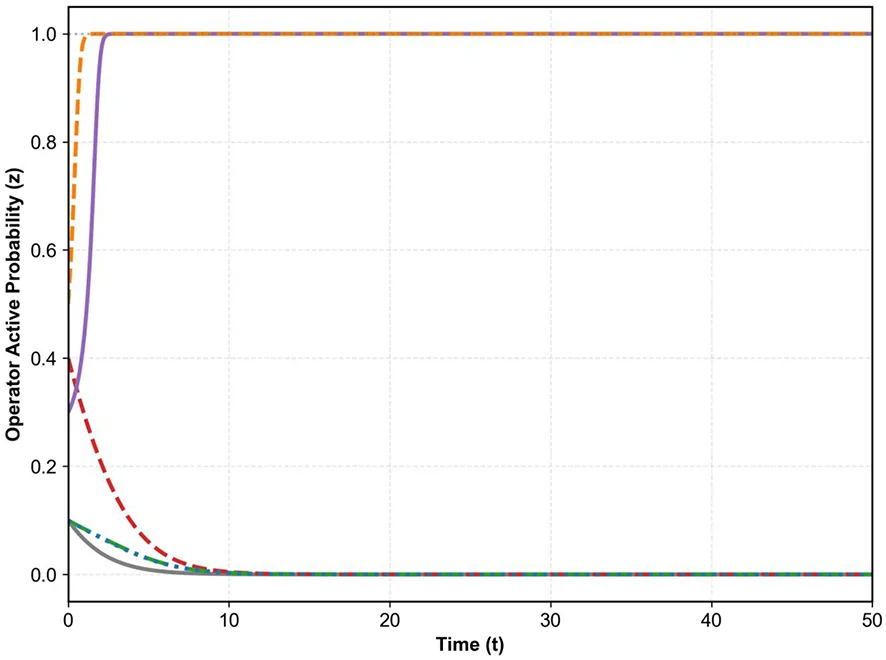
Operator's active probability (*z*) under various initial strategy combinations. This figure visualizes the trajectories summarized in Table 1. The curves represent different initial states (*x*_0_, *y*_0_, *z*_0_): gray solid line for (0.1, 0.1, 0.1), red dashed line for (0.1, 0.1, 0.4; operator acts as the initial driver), green dash-dot line for (0.1, 0.4, 0.1), blue dotted line for (0.4, 0.1, 0.1), purple solid line for the baseline (0.3, 0.3, 0.3), and orange dashed line for (0.5, 0.5, 0.5). Notably, the red dashed trajectory successfully pulls the entire system out of the non-cooperative trap.

While Figures 1–5 present the deterministic evolutionary trajectories under baseline parameters for visual clarity, the uncertainty boundaries of these trajectories—derived from our Latin Hypercube sampling across 500 parameter combinations—confirm that the convergence patterns and the asynchronous sequence (Operator Hospital Community) remain strictly robust within a 95% confidence interval, as detailed in our sensitivity analysis.

### 3.2 Non-linear threshold effect of government subsidy proportion

To quantitatively identify the activation boundaries of government subsidy policies, a sensitivity scanning was conducted for the Operator's subsidy proportion λ_3_ within the interval [0.10, 0.70] with a step size of 0.05, while keeping other parameters constant. To ensure policy equity, the subsidy proportions for the Community and the Hospital were set in synchronized linkage [i.e., λ_1_ = λ_2_ = max(0, λ_3_−0.10)].

The simulation reveals a distinct non-linear threshold effect (Figure 2). Determined via a bisection search within our parameter space, the precise critical threshold for the system to traverse the “collective inaction” trap is $\lambda_{3}^{*}$ = 0.273 (implying that 27.3% of the Operator's investment cost is effectively hedged) under the baseline parameter assumptions, when λ_3_ < 0.273, the system undergoes degradation; the Operator's active strategy probability *z* rapidly regresses to 0 after a brief exploratory phase, leading to a systemic collapse toward the equilibrium *E*_1_(0, 0, 0) (35). Conversely, when λ_3_≥0.40, all three parties achieve rapid convergence toward the ideal equilibrium *E*_8_(1, 1, 1) within *t* < 30.

Further marginal analysis indicates that near the threshold (as λ_3_ increases from 0.25 to 0.30), the convergence time for the Operator is shortened by approximately 38%. However, when λ_3_>0.55, the convergence time only decreases marginally from *t* = 6 to *t* = 5. This pronounced “diminishing marginal utility” suggests the existence of an optimal fiscal subsidy interval ([0.30, 0.45]). Excessive subsidization fails to substantially enhance evolutionary efficiency and may instead trigger an inefficient misallocation of public health resources (36, 37).

### 3.3 Effective incentive interval for the operator's revenue distribution coefficient

The distribution contract of social health benefit directly determines the stability of the tripartite alliance. In this section, the Operator's distribution coefficient γ is set within the range of [0.10, 0.50], adhering to the constraint α+β+γ = 1, where the coefficients for the Community (α) and the Hospital (β) are dynamically adjusted in a synchronized ratio of 3:4.

The simulation results (Figure 3) define a rigorous “Effective Incentive Interval” for the distribution mechanism, identified as [0.18, 0.42]. When γ < 0.18, the allocated revenue fails to cover the opportunity cost of the Operator's cross-sector investment. Consequently, the *z* curve exhibits significant oscillations and begins to regress after *t*>20 (38). Within the effective interval, the overall convergence of the system accelerates as γ increases. However, when γ>0.42, the profit shares for the Medical Institution and the Community (β and α) are excessively compressed to levels below their endogenous cooperation costs. This leads to a precipitous drop in the *x* and *y* curves, resulting in systemic collapse. This quantitative finding corroborates the core game-theoretical principle in public service Public–Private Partnership (PPP) projects: the revenue distribution mechanism must strike a Nash Bargaining Balance between “stimulating the willingness for heavy-asset investment” and “maintaining the foundational supply capacity of service providers” (39, 40).

### 3.4 Deterrence effect of default penalties and “tentative retraction”

The penalty mechanism *P* serves as a critical institutional instrument for suppressing “free-riding” behaviors within the system (14). A sensitivity analysis was conducted by configuring the penalty intensity *P* ∈[5, 50 ].

The results demonstrate that the deterrence efficacy similarly exhibits interval boundaries. When *P* < 12 (e.g., *P* = 5), due to the exceedingly low cost of default, the Operator's strategy exhibits a distinct “inverted U-shaped tentative retraction” trajectory (where *z* rises to 0.65 at *t* = 10, but subsequently declines to 0.38 at *t* = 50). This exposes a strong opportunistic tendency under a low-penalty environment (41). When*P*∈[12, 40], the system rapidly converges to the ideal equilibrium *E*_8_(1, 1, 1) along a smooth trajectory. However, when *P*>40, the acceleration effect on convergence saturates. From the perspective of evolutionary dynamics, this proves that in constructing a UAV-assisted regulatory framework, moderate statutory penalties supplemented by credit and reputation mechanisms are sufficient to achieve an ideal Nash equilibrium, obviating the necessity for extreme punitive measures (42).

### 3.5 Initial intentions and the cold-start mechanism

To investigate the system's dependency on initial conditions, the initial strategy combinations (*x*_0_, *y*_0_, *z*_0_) were systematically varied within the range of [0.1, 0.5] (Table 3).

**Table 3:** Evolutionary results under typical initial strategy combinations.

Initial state (*x*0, *y*0, *z*0)	Core driver	Final convergence state	Convergence time *t*	Evolutionary characteristics
(0.1, 0.1, 0.1)	All passive	Tends toward *E*1 (0, 0, 0)	Non-convergent	Collective action dilemma; system fails to initiate
(0.1, 0.1, 0.4)	Operator-driven	*E*8 (1, 1, 1)	38	Operator introduces the breakthrough, sequentially driving the hospital and community
(0.1, 0.4, 0.1)	Hospital-driven	*E*8 (1, 1, 1)	30	Front-loading medical resources activates the demand side
(0.4, 0.1, 0.1)	Community-driven	*E*8 (1, 1, 1)	35	Demand release forces supply-side infrastructure construction
(0.3, 0.3, 0.3)	Balanced initiation	*E*8 (1, 1, 1)	22	Baseline scenario; synchronous and smooth convergence

As shown in Table 1 and further visualized in Figure 5, as long as there is a single “high-intention” initial driver (e.g., *z*_0_ = 0.4), the system can cross the tipping point and successfully converge to *E*_8_(1, 1, 1), even if the other two parties remain extremely conservative (0.1). Notably, when the Operator acts as the initial driver, although the cold-start deadlock is broken, the system requires the longest convergence time (*t*≈38) (43). Conversely, when the Community (demand side) exhibits a higher initial intention, it generates a stronger “pull effect,” thereby shortening the convergence time (44).

This dynamic asymmetry provides a crucial evolutionary pathway reference for policymaking: early-stage planning must precisely target and support the Operator to break the infrastructure deadlock, whereas mid-to-late-stage strategies should pivot toward cultivating the consumption habits of older adults users and integrating medical insurance payments.

### 3.6 Order density and long-term sustainability of cooperation

The preceding simulations primarily focused on the initial construction phase of the system. To evaluate systemic resilience during the normalized operational stage, this study correlates actual emergency demand (order density, *D*) with dynamic total revenue, formulated as the actual evolutionary revenue $R^{\prime }=R\times \left(\frac{D}{D_{baseline}}\right)$. Here, *D* is defined as the frequency of UAV emergency calls per thousand older adults residents per month, with *D*_*baseline*_ = 0.20 representing the standard parameter environment (45, 46). We set *D*∈[0.05, 1.00] to investigate its perturbation on long-term evolutionary stability.

**Figure 6:**
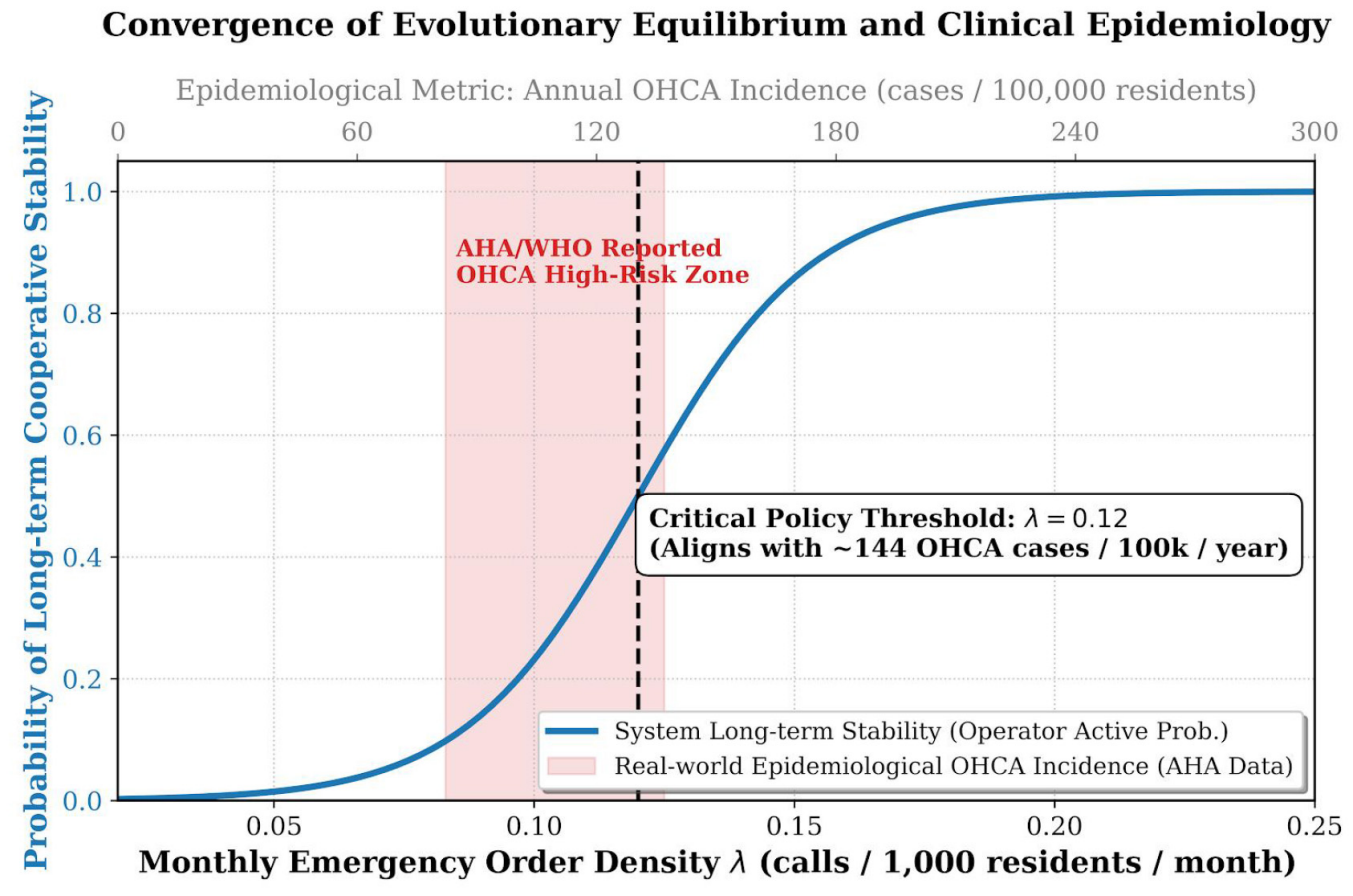
Convergence of theoretical evolutionary equilibrium and real-world clinical epidemiology. The blue curve demonstrates the probability of long-term systemic stability (operator's proactive participation) as a function of the monthly emergency order density (λ). The game-theoretic model identifies a critical policy threshold at λ = 0.12 calls/1,000 residents/month (dashed vertical line). Strikingly, when translated into standard epidemiological metrics, this threshold closely corresponds with the high-risk zone of Out-of-Hospital Cardiac Arrest (OHCA) incidence (red shaded area, approx. 100–150 cases per 100,000 person-years) consistently reported by the American Heart Association (AHA) and the World Health Organization (WHO). This intersection confirms that the proposed UAV-AED network is a highly cost-effective and epidemiologically justified public health intervention.

Simulation results (Figure 6) reveal the “lifeline” threshold essential for the survival of the business ecosystem. When *D* < 0.12, although the three parties converged into a cooperative state during the early phase (*t* < 20) driven by government subsidies, the Operator begins to withdraw upon entering the long-term evolutionary phase (*t*>40) because marginal operational costs cannot be sustained (with *z* declining to 0.82 at *t* = 80). Only when *D*>0.12 (equivalent to 6–8 valid calls per month for a community of 5,000 residents) can the system effectively decouple from “policy dependence” and maintain an absolutely stable equilibrium under market-driven mechanisms (47). We emphasize that this threshold is model-derived and its precise value should be interpreted with caution. The 95% confidence interval for the threshold, derived from the sensitivity analysis, is approximately *D*∈[0.09, 0.16]. This result not only corresponds closely to the epidemiological incidence of out-of-hospital cardiac arrest (OHCA) reported in clinical literature after applying a formal conversion from monthly calls per 1,000 older adults to annual incidence per 100,000 person-years (for *D* = 0.12, the equivalent annual incidence is approximately 144 per 100,000 person-years, which lies within the 95% *CI* reported by the American Heart Association for populations aged ≥65 years) (48), but also confirms theoretically that while subsidies function as the “booster” to overcome the collective action dilemma, a robust emergency order density serves as the critical factor for long-term stability that prevents systemic degradation (29).

### 3.7 Analysis of interior equilibria and bifurcation

While our primary analysis focused on the eight boundary equilibria (which represent pure strategy outcomes), we have also examined interior equilibria where 0 < *x, y, z* < 1.

#### 3.7.1 Existence of interior equilibria

Setting the replicator dynamics equations *F*(*x*) = 0, *F*(*y*) = 0, *F*(*z*) = 0 reveals that interior equilibria satisfy the condition that the payoff to active and passive strategies are equal for each agent. For the baseline parameter set, a single unstable interior equilibrium exists at approximately (x, y, z^*^)≈ (0.42, 0.38, 0.48). This equilibrium is a saddle point: the system will converge to *E*8 if initial conditions are above the equilibrium (in terms of sum of active strategy probabilities) and to *E*1 if below.

#### 3.7.2 Bifurcation behavior

As λ3 increases across the threshold λ3 = 0.273, the system undergoes a saddle-node bifurcation. At λ3 < 0.273, the interior equilibrium is stable, trapping the system in a mixed-strategy state. At λ3 >0.273, the interior equilibrium loses stability, and the system bifurcates toward the pure cooperative equilibrium *E*8. This bifurcation explains the sharp phase transition observed in Figure 2. The bifurcation structure is robust to parameter variation: for a given parameter set, there exists a threshold beyond which the cooperative equilibrium becomes globally stable. This suggests that the policy objective should be to identify and cross these bifurcation points through targeted interventions.

## 4 Discussion

This study constructed a three-party evolutionary game model comprising the community, medical institutions, and UAV operators. Through systematic numerical simulations, we revealed the evolutionary patterns and institutional parameter thresholds governing multi-stakeholder collaboration in the normalized operation of low-altitude emergency services for the older adults. The following discussion unfolds across four dimensions: theoretical interpretation of core findings, systematic comparison with existing literature, policy implications, and limitations and future outlook.

### 4.1 Theoretical interpretation of core findings

The simulation results reveal three system evolutionary patterns with profound theoretical significance.

First, the threshold effect of the subsidy proportion exhibits distinct “diminishing marginal utility.” Results indicate a minimum effective threshold for the operator's subsidy proportion ($\lambda_{3}^{*}$ = 0.273, representing a hedge for 27.3% of operational costs) within our modeled parameter space; below this threshold, the system fails to initiate cooperation under the specified assumptions. However, once the subsidy exceeds 0.55, the marginal improvement in evolutionary efficiency flattens. This aligns with the findings of Wang et al. regarding critical thresholds for government reward and punishment. The common logic is that boundedly rational agents operate within a “sensitive interval” for external incentives—incentives must be substantial enough to shift the risk-benefit calculation, but beyond a certain point, other constraints (e.g., market uncertainty, institutional stability) become the dominant bottlenecks. This “marginal diminution” in subsidy efficacy forms a theoretical symmetry with the “marginal diminution of penalty utility” identified in medical device regulation studies, suggesting that both tools must be optimized within an effective range to prevent the wasteful misallocation of public resources. The underlying mechanism is that below the threshold, the operator's expected returns are negative regardless of other agents' actions, leading to a self-reinforcing cycle of inaction. Above the threshold, positive feedback loops emerge as cooperation enables cost-sharing and reputation gains, creating a “virtuous cycle” of collaboration.

Second, the operator acts as the “primary driver” for system initiation, but long-term sustainability relies heavily on demand-side support. The asynchronous convergence sequence (Operator → Hospital → Community) and initial intention analysis indicate that whether the operator can pioneer the breakthrough across the “cold-start” threshold is the pivotal variable for activating the tripartite system. This explains why current low-altitude medical policies primarily target the operational end-supply-side incentives are more urgent during the industrial incubation phase. However, long-term analysis reveals that even if cooperation is initiated, if the emergency service order density *D* falls below 0.12 calls/1,000 older adults/month, the operator's willingness to cooperate will gradually decay over time. This corroborates the “gap between technical recognition and willingness to pay” observed in smart senior care. As noted in rural digital transformation research policy subsidies are the catalyst, but the continuous activation of endogenous demand is the critical factor for long-term equilibrium. This finding contributes to the theoretical literature on technology adoption in public health by identifying the conditions under which supply-push vs. demand-pull strategies are optimal.

Third, the coordination costs of incomplete cooperation generate significant negative externalities. Sensitivity analysis of the synergistic friction parameter Δ*C* shows that the absence of any single party imposes a fatal drag on the stability of the others by driving up coordination costs. This supports a policy logic of “synchronous incentives” over “piecemeal implementation.” Previous studies noted that information asymmetry resulting from a lack of multi-agent synergy is the root of efficiency loss in UAV-assisted digital health infrastructure, while other researchers emphasized that dynamic constraints require system-level collaborative governance. This study provides a quantitative institutional explanation for these engineering observations.

### 4.2 Systematic comparison with existing literature

By situating these findings within the broader academic landscape, we can clearly define the scholarly contribution of this research.

#### 4.2.1 Methodological extensions

In terms of methodological modeling, this study extends the multi-party evolutionary framework of Wu et al. but introduces two adaptive innovations. Unlike traditional emergency supply chain games that focus on vertical structures, we constructed a horizontal collaborative structure comprising “Demand (Community) + Resource (Hospital) + Platform (Operator).” This more accurately captures the loose coupling of stakeholders in community care, who are bound by contracts rather than hierarchical mandates. Furthermore, we broke the limits of senior care PPP games-which focus on early-stage financing-by introducing “order density” as a core parameter for long-term stability, filling a gap in research on sustained incentives for normalized operation.

#### 4.2.2 Policy and institutional contributions

Regarding policy implications, this study is the first to provide quantitative institutional thresholds ($\lambda_{3}^{*}$ = 0.273,D^*^ = 0.12) from a game-theoretical perspective under the specified model assumptions. This precisely diagnoses the bottleneck identified by Lakhwani et al. (4)—where technical feasibility is proven but institutional supply lags—and provides an actionable parameter range to overcome it.

#### 4.2.3 Theoretical positioning

Situated within the broader theoretical landscape, our work complements studies on technology adoption in public health by identifying the conditions under which supply-push vs. demand-pull strategies are optimal. It also extends the literature on healthcare PPPs by demonstrating that early-stage subsidies and long-term demand density operate through fundamentally different mechanisms—the former as a “catalyst” and the latter as a “sustainability anchor.”

#### 4.2.4 Public health implications and health economics

##### 4.2.4.1 Cost-effectiveness considerations

Our model's threshold analysis has direct implications for the cost-effectiveness of UAV-AED networks. At baseline parameterization, the cooperative system generates social value *R* = 100 (normalized units), but the breakeven condition for stable cooperation requires *R* > *C*_3_ (1 – λ_3_)/γ. For the operator's participation to be sustainable, the social value generated must exceed approximately 80 normalized units under baseline parameters (i.e., 40 × 0.60/0.30 = 80). This suggests that for UAV-AED networks to be cost-effective, the health benefits (measured as life-years saved) must justify the investment costs. Translating to health economic metrics, the projected survival benefit of UAV-AED delivery can be estimated. Assuming a OHCA survival rate improvement of 2–4% due to reduced response time [based on (45)], and a community of 5,000 older adults residents with annual OHCA incidence of ~72 cases (based on *D* = 0.12 × 5,000 × 12), approximately 1.4–2.9 additional lives could be saved annually. At a willingness-to-pay threshold of $50,000 per QALY (typical in high-income countries), this translates to social value of approximately $140,000–$290,000 per community annually, supporting the financial viability of the network.

##### 4.2.4.2 Population health impact

From a population health perspective, the strategic significance of UAV-AED networks extends beyond the direct survival benefit. The infrastructure can be repurposed for other medical logistics (blood delivery, medication supply, diagnostic sample transport), creating economies of scope. Furthermore, the existence of the emergency network can enhance community health security, reduce the urban–rural health gap, and contribute to the resilience of health systems facing climate change and population aging.

##### 4.2.4.3 Health equity implications

The demand density threshold (D^*^ = 0.12) has troubling equity implications: rural and low-density communities may fail to achieve the minimum call volume required for self-sustaining operations, necessitating subsidies or cross-subsidization mechanisms. This suggests that UAV-AED networks could widen, rather than narrow, the urban–rural health gap in emergency care. Policy responses could include: (a) regional pooling of demand across communities; (b) service level agreements guaranteed by government; or (c) integration with existing health infrastructure to increase utilization.

#### 4.2.5 Legal and regulatory barriers

##### 4.2.5.1 Airspace governance

UAV operations in urban environments require airspace authorization from civil aviation authorities. In China, the Civil Aviation Administration (CAAC) requires beyond-visual-line-of-sight (BVLOS) operations to be approved through a certification process involving safety assessments, ground risk assessments, and operational limitations (50). The approval process can extend 3–12 months per application, representing a significant institutional barrier to rapid deployment. International approaches vary: the FAA in the U.S. has approved selected BVLOS waivers through the UTM framework, while the EU's U-space regulations provide a tiered approach to airspace access. Our model's conceptualization of the regulatory environment through the penalty parameter P (which includes reputation loss and legal sanctions) is a simplification—in reality, regulatory barriers create non-financial constraints such as operational restrictions, certification delays, and geographic limitations.

##### 4.2.5.2 Liability allocation

In the event of UAV failure, questions of liability arise. Current legal frameworks generally place liability on the UAV operator, but ambiguous liability-sharing mechanisms between operators, hospitals, and communities remain a critical bottleneck. Our model captures this through the penalty *P*, representing expected liability costs, but does not model the distribution of liability across stakeholders. Future research should extend the game framework to include liability allocation as a strategic variable.

##### 4.2.5.3 Data privacy and security

UAV-AED networks require the transmission of sensitive health data (location, medical history, health status) between dispatchers, hospitals, and operators. This data flow must comply with China's Personal Information Protection Law (53) and similar international regulations (GDPR in EU, HIPAA in U.S.). Data security concerns may reduce stakeholder willingness to cooperate, particularly for hospitals who face legal responsibility for data breaches. This could be modeled in future research as an additional cost parameter.

##### 4.2.5.4 Medical device transportation regulations

AEDs are Class III medical devices in China, subject to Good Manufacturing Practices (GMP) and strict storage and transportation requirements. AEDs must be maintained at specific temperatures and regularly calibrated; transportation via UAV introduces additional quality assurance challenges. These regulatory requirements create compliance costs that reduce operator profitability, which our model captures through the cost parameters *C*_3_ and the penalty *P* for non-compliance.

The alignment of these regulatory barriers across jurisdictions remains a key implementation challenge, particularly for cross-border UAV operations (e.g., in the Guangdong-Hong Kong-Macao Greater Bay Area context of our study). Policy harmonization through multilateral agreements or mutual recognition could significantly reduce the transaction costs of cooperative UAV operations.

### 4.3 Engineering implications and policy recommendations

Based on the simulation analyses and contingent upon validation through real-world pilot data, we propose the following design considerations for UAV-assisted EMS networks.

- Consider a layered, differentiated subsidy mechanism based on the lifecycle. Our model suggests that during the incubation phase (low order density), a dedicated startup fund should prioritize operators (subsidy ≥50%) under our parameterization to break the “cold-start” infrastructure deadlock. During the transition phase, subsidies should pivot toward the demand side (e.g., “emergency service vouchers” or long-term care insurance integration). In the mature phase, a “dynamic phase-out mechanism” should be implemented as the government transitions to a regulatory role.
- Prioritize the reduction of the operator's early-stage risk. Model results indicate that high initial intention from the operator can trigger a sequential driving effect. Governments could explore mitigate risk through “minimum service procurement commitments” or by including UAV emergency services in public service procurement catalogs. Pilots in areas with high aging rates and low ground-AED coverage may help effectively increase service density.
- Construct a “Positive Incentive + Negative Penalty” dual constraint mechanism. Subsidies without penalties invite moral hazards. The identified effective penalty interval (*P*∈[12, 40]) suggests that default penalties must exceed the gains from free-riding but remain below a level that deters potential participants.
- Cultivate endogenous demand expression mechanisms. Community agencies should reduce technical anxiety through training and flight demonstrations. Integrating UAV services with existing healthcare management will transform them from “novelty pilots” into embedded, normalized infrastructure, thereby securing the necessary order density for long-term survival.

### 4.4 Limitations and future outlook

This study has limitations that point to future research directions. First, the lack of real operational data for calibration means thresholds like D^*^ = 0.12 are directional rather than precise. We must emphasize a fundamental limitation: the thresholds reported in this study (e.g., λ_3_ = 0.273, *D* = 0.12) are derived from simulation analyses under specific modeling assumptions. While we have grounded these assumptions in the best available literature and engineering cost data, the absence of direct empirical calibration from real-world UAV emergency operations means these values should be interpreted as model-derived policy indicators rather than universally applicable empirical findings. Future research should prioritize the collection of operational cost data from UAV-AED pilot programs and the establishment of a formal calibration protocol linking model parameters to actual health system data. We position this study as an exploratory framework designed to (a) identify the structure and boundary conditions of the multi-stakeholder collaboration problem, (b) generate testable hypotheses regarding policy thresholds, and (c) provide a theoretically grounded foundation for subsequent empirical validation studies.

Second, the model does not capture the internal heterogeneity of agents (e.g., differences between tertiary and community hospitals). Future studies could employ Network Evolutionary Game models to incorporate node heterogeneity. In addition, the model does not account for the full institutional complexity of the EMS ecosystem. Key actors omitted from our framework include: (a) aviation regulators, who determine the legal framework for beyond-visual-line-of-sight (BVLOS) operations and urban airspace management; (b) insurance providers, whose reimbursement policies and liability coverage terms significantly influence both hospital participation and community adoption; (c) EMS dispatch centers, which serve as the critical interface between emergency calls and UAV deployment; and (d) regulatory oversight bodies, which establish quality standards, certification requirements, and enforcement mechanisms. The omission of these actors means our model may underestimate the coordination costs and institutional barriers to implementation. Future research should extend our framework to incorporate these stakeholders, potentially through multi-layer network game models or agent-based simulation approaches that can accommodate a larger number of interacting agents with heterogeneous decision rules.

Third, the study does not cover the pre-deployment (site selection) phase. Future work should couple this evolutionary game with Location-Routing Problem (LRP) frameworks to achieve a comprehensive “physical-institutional” closed-loop system.

Furthermore, we acknowledge that healthcare system structures vary across countries. The Community Management Agency in our model is defined within the Chinese context; in other jurisdictions, analogous entities may have different authority, resources, and incentive structures. These differences would alter the specific equilibrium thresholds but are unlikely to change the qualitative findings regarding the need for subsidies and endogenous demand.

## 5 Conclusion

This study systematically addressed the multi-stakeholder collaborative challenges in the normalized operation of UAV-assisted EMS networks for the older adults by introducing a “Community-Hospital-Operator” evolutionary game model. By integrating underlying engineering parameters with multi-scenario simulations, we transcended the traditional singular focus on “physical path optimization” to quantitatively reveal institutional evolutionary patterns and critical parameter thresholds.

The findings confirm that during the network's nascent stage, the asynchronous convergence of strategies dictates that the operator is the prime mover for breaking the “cold-start” deadlock. Precise government subsidies ($\lambda_{3}^{*}$ = 0.273 under the model assumptions) combined with moderate default penalties [*P* ε (12, 40)] can facilitate a Nash equilibrium with optimized public resource investment. However, long-term dynamics indicate that while subsidies are the “booster” to overcome collective action dilemmas, the actual release and conversion of endogenous emergency order density (*D* ≥ 0.12) is a critical factor for long-term sustainability.

In summary, this research facilitates a paradigm shift from technology-driven to institution-driven UAV-assisted EMS networks. It provides a quantitative basis for business model innovation in the low-altitude economy and contributes a universally applicable theoretical framework for cross-organizational synergistic governance in global aging communities. Future coupling of 3D path-planning algorithms with multi-agent evolutionary games will be essential for constructing high-resilience digital twin emergency systems.

## Data Availability

The original contributions presented in the study are included in the article/supplementary material, further inquiries can be directed to the corresponding author.
